# Functional Characterization of the m^6^A-Dependent Translational Modulator PfYTH.2 in the Human Malaria Parasite

**DOI:** 10.1128/mBio.00661-21

**Published:** 2021-04-27

**Authors:** Ameya Sinha, Sebastian Baumgarten, Amy Distiller, Emma McHugh, Patty Chen, Meetali Singh, Jessica M. Bryant, Jiaqi Liang, Germano Cecere, Peter C. Dedon, Peter R. Preiser, Stuart A. Ralph, Artur Scherf

**Affiliations:** a School of Biological Sciences, Nanyang Technological University, Singapore, Republic of Singapore; b Antimicrobial Resistance Interdisciplinary Research Group, Singapore-MIT Alliance for Research and Technology, Singapore, Republic of Singapore; c Biology of Host-Parasite Interactions Unit, Department of Parasites and Insect Vectors, Institut Pasteur, Paris, France; d CNRS, ERL 9195, Paris, France; e INSERM, Unit U1201, Paris, France; f Department of Biochemistry and Molecular Biology, Bio21 Molecular Science and Biotechnology Institute, The University of Melbourne, Parkville, Australia; g Mechanisms of Epigenetic Inheritance, Department of Developmental and Stem Cell Biology, Institut Pasteur, Paris, France; h CNRS, UMR 3738, Paris, France; i Department of Biological Engineering, Massachusetts Institute of Technology, Cambridge, Massachusetts, USA; University of Georgia

**Keywords:** *Plasmodium falciparum*, m^6^A mRNA methylation, translational repression, PfYTH, malaria parasite

## Abstract

Posttranscriptional regulation of gene expression is central to the development and replication of the malaria parasite, Plasmodium falciparum, within its human host. The timely coordination of RNA maturation, homeostasis, and protein synthesis relies on the recruitment of specific RNA-binding proteins to their cognate target mRNAs. One possible mediator of such mRNA-protein interactions is the N^6^-methylation of adenosines (m^6^A), a prevalent mRNA modification of parasite mRNA transcripts. Here, we used RNA protein pulldowns, RNA modification mass spectrometry, and quantitative proteomics to identify two P. falciparum YTH domain proteins (PfYTH.1 and PfYTH.2) as m^6^A-binding proteins during parasite blood-stage development. Interaction proteomics revealed that PfYTH.2 associates with the translation machinery, including multiple subunits of the eukaryotic initiation factor 3 (eIF3) and poly(A)-binding proteins. Furthermore, knock sideways of PfYTH.2 coupled with ribosome profiling showed that this m^6^A reader is essential for parasite survival and is a repressor of mRNA translation. Together, these data reveal an important missing link in the m^6^A-mediated mechanism controlling mRNA translation in a unicellular eukaryotic pathogen.

## Abstract (precis)

Infection with the unicellular eukaryotic pathogen Plasmodium falciparum causes malaria, a mosquito-borne disease affecting more than 200 million and killing 400,000 people each year. Underlying the asexual replication within human red blood cells is a tight regulatory network of gene expression and protein synthesis.

## INTRODUCTION

The unicellular apicomplexan parasite Plasmodium falciparum is the causative agent of the most virulent form of human malaria, a mosquito-borne infectious disease which remains a global health threat (1). All symptoms of the disease are caused by the asexual replication of the parasite within human red blood cells (RBCs). During the 48-h intraerythrocytic developmental cycle (IDC), each parasite replicates and creates up to 32 daughter cells that reinfect new RBCs. During the IDC, monocistronic gene expression is tightly controlled at least in part by a family of apicomplexan-specific ApiAP2 transcription factors and epigenetic coactivators (2–4). The mRNAs of a gene reach peak abundance only once per cycle (5), which corresponds to a time preceding the period when the encoded protein is most required. Selective silencing of subtelomeric and central chromosomal regions by heterochromatin further orchestrates the timely expression of variant surface antigens (6) and sexual commitment (7, 8). In addition to epigenetic and transcriptional control of mRNA abundances, extensive modulation of mRNA stability and translational efficiency occurs throughout the IDC (9–11). However, a mechanism mediating such posttranscriptional control on a transcriptome-wide level remained unknown.

Recently, N^6^-methylation of adenosines (m^6^A) at internal positions of mRNA transcripts has been identified as the most abundant mRNA modification in P. falciparum (12). Due to the exceptionally high adenosine content of the parasite’s mRNA transcriptome (∼45%), global m^6^A levels exceed those measured among other eukaryotes. In addition, m^6^A levels are highly dynamic and double over the course of the IDC, suggesting m^6^A is the basis of a key posttranscriptional control mechanism in the parasite. Similar to what has been observed in model eukaryotes (13), m^6^A methylation in P. falciparum inversely correlates with mRNA stability. However, m^6^A-modified mRNAs tend to also show lower translational efficiencies (12), suggesting a new m^6^A-mediated phenotype that has so far not been described in eukaryotes.

**FIG 1:**
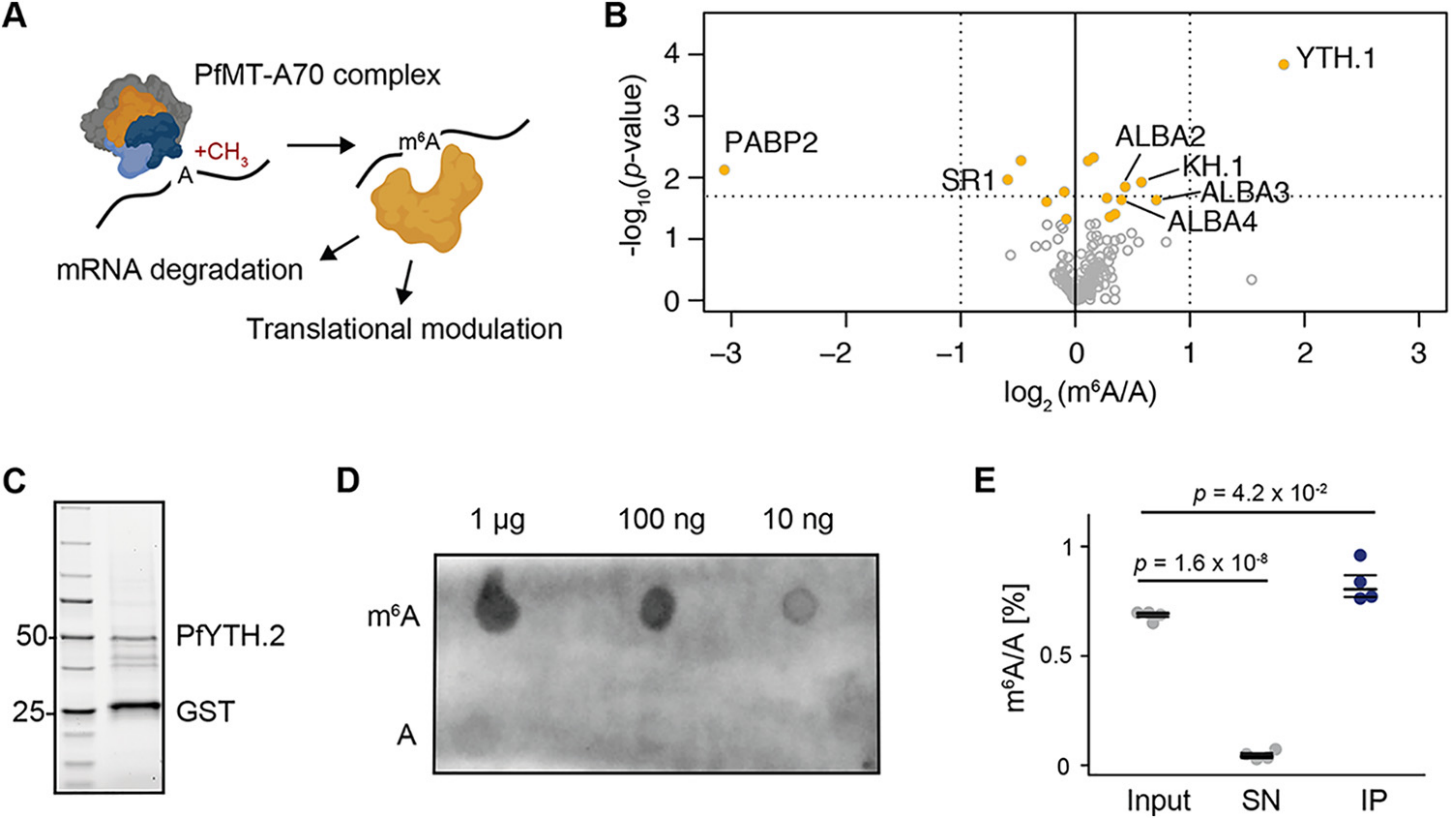
PfYTH.1 and PfYTH.2 are m^6^A-binding proteins in P. falciparum. (A) Model for the mode of action of the PfMT-A70 m^6^A methylation complex and putative m^6^A-binding “reader” proteins mediating mRNA degradation and translational repression in P. falciparum. (B) TMT-based quantification of proteins that were preferentially pulled down *in vitro* either with an m^6^A-methylated (right half) or unmethylated (left half) RNA (see also Fig. S1B in the supplemental material). The *x* axis represents the average intensity of the TMT reporter ion in the m^6^A-methylated oligonucleotide pulldown over those in the unmethylated oligonucleotide pulldown. (C) Stain-free gel of recombinant PfYTH.2 with an N-terminal glutathione *S*-transferase (GST) tag. The lower band corresponds to free GST. Numbers on the left show size in kilodaltons. The GST-PfYTH.2 fusion protein is detectable at ∼50 kDa (calculated weight, 60.1 kDa). (D) Dot blot assay showing binding of recombinant PfYTH.2 to m^6^A-methylated RNA oligonucleotides (Fig. S1B) in a concentration-dependent manner (top, m^6^A) but no binding to the identical RNA oligonucleotide without m^6^A (bottom, A). Concentrations of spotted RNA oligonucleotide are indicated above the plot. Signal was obtained using an anti-GST antibody (GE Healthcare 27457701). (E) LC-MS/MS measurements of m^6^A/A ratios in native P. falciparum mRNA before (input) and after (supernatant [SN]) incubation with recombinant PfYTH.2 protein (IP). m^6^A is depleted in the non-PfYTH.2-bound fraction collected after incubation and enriched in the PfYTH.2-bound fraction. *P* values were calculated with a two-sided independent-samples *t* test. Average and median values are shown for four individual experiments, represented as dots.

To achieve a functional outcome, m^6^A recruits specific “reader” proteins (14). Such proteins, especially those containing a YT521-B homology (YTH) domain, have been identified as evolutionarily conserved in many eukaryotes (Fig. 1A) and can affect a multitude of cellular processes via modulation of mRNA degradation and alternative splicing as well as the function of noncoding RNAs (15). In addition to an evolutionarily conserved m^6^A core “writer” complex, P. falciparum harbors two proteins that contain a YTH domain: PfYTH.1 (PF3D7_1419900) and PfYTH.2 (PF3D7_0309800) (12). During the IDC, these two genes reach peak transcription in the late ring and early trophozoite stages (i.e., 16 to 20 h postinfection [hpi]). However, while PfYTH.1 is exclusively expressed during the IDC, PfYTH.2 is transcribed at even higher levels in transmission stages (i.e., gametocytes and sporozoites) (Fig. S1A). Outside their conserved YTH domain, PfYTH.1 and PfYTH.2 are highly divergent (12), suggesting that the two proteins have distinct functions. However, the m^6^A-binding ability of these PfYTH proteins or their specific roles in dictating the functional outcome of m^6^A in P. falciparum remain unknown.

10.1128/mBio.00661-21.1 FIG S1 (A) Transcript levels (reads per kilobase of exon per one million mapped reads [RPKM]) of PfYTH.1 (grey) and PfYTH.2 (blue) during the IDC (hours postinvasion) and in gametocytes (G), oocysts (O), and salivary gland sporozoites (S). (B) Sequence of the synthesized RNA oligonucleotides consisting of five repeats of the m^6^A consensus motif (GGACA) either with (top) or without m^6^A (bottom) at the central adenosine motif position. (C) Phylogenetic tree of PfYTH proteins and known m^6^A-binding YTH proteins from other eukaryotes. Numbers on nodes denote bootstrap values. (D) Domain architecture of PfYTH.1 and PfYTH.2 compared to known m^6^A-binding YTH proteins. Domain annotations are derived from the InterPro database and intrinsically disordered regions (IDR) from D2P2 (51). (E) Detailed alignment of the YTH domain from P. falciparum and known m^6^A-binding YTH proteins found in other eukaryotes. Sites of mutated recombinant PfYTH.2 are indicated with a red X (W46 and D143). For panels C, D and E, Pf, P. falciparum; Hs, Homo sapiens; Dr, Danio rerio; Dm, Drosophila melanogaster; Atha, Arabidopsis thaliana; Sc, Saccharomyces cerevisiae; Bs, Bodo saltans. (F) Western blot of recombinant PfYTH.2 with an N-terminal GST tag using an anti-GST antibody. Numbers on the left indicate protein size in kilodaltons. (G) Dot blot assay as in Fig. 1D but for recombinant PfYTH.2 with mutations W46L (top) and D143E (bottom). Location of the mutation indicated with a red X in panel D. Concentrations of spotted RNA oligonucleotide are indicated above the plot. Signal was obtained using an anti-GST antibody (GE Healthcare 27457701). (H) Workflow of the *in vitro* mRNA pulldown using recombinant PfYTH.2 protein (yellow) immobilized on GST magnetic beads (purple) and subsequent proteomic analysis. (I) LC-MS/MS measurements of m^7^G/G ratios in native P. falciparum mRNA before (input) and after (supernatant [SN]) incubation with recombinant PfYTH.2 (immunoprecipitate [IP]). Average and median values are shown for four individual experiments, represented as dots. Download FIG S1, PDF file, 0.7 MB. Copyright © 2021 Sinha et al. 2021 Sinha et al. https://creativecommons.org/licenses/by/4.0/ This content is distributed under the terms of the Creative Commons Attribution 4.0 International license.

Here, using RNA modification mass spectrometry and RNA-mediated protein immunoprecipitation combined with quantitative proteomics, we demonstrate that PfYTH.1 and PfYTH.2 are m^6^A-binding proteins. We show that PfYTH.2 is essential for normal parasite growth and associates with the translational machinery during the IDC. Inducible knock sideways followed by ribosome profiling showed that PfYTH.2 is likely a repressor of mRNA translation, revealing a role for m^6^A mRNA methylation in the fine-tuning of gene expression in P. falciparum.

## RESULTS

### Identification of PfYTH proteins as m^6^A “readers”.

To identify putative m^6^A-binding proteins in the P. falciparum proteome, we first performed an unbiased protein pulldown using a synthetic RNA oligonucleotide containing five repetitions of the consensus m^6^A motif (GGm^6^ACA) and a nonmethylated control RNA oligonucleotide of identical sequence and length but without m^6^A methylation (i.e., 5 repetitions of GGACA) (see Fig. S1B in the supplemental material). Quantitative tandem mass tag (TMT) proteomics identified PfYTH.1 (PF3D7_1419900) as the most significantly enriched protein in the pulldown with the methylated RNA oligonucleotide compared to that with the nonmethylated RNA oligonucleotide. Other proteins that were significantly enriched in the methylated oligonucleotide pulldown included three of the four ALBA DNA/RNA-binding proteins (ALBA2 to -4) (Fig. 1B; see also Table S1). In addition, we found another putative RNA-binding protein (PF3D7_0605100) to be significantly more abundant in the methylated oligonucleotide pulldown. This protein encodes four K homology (KH) domains and was named PfKH.1. In contrast, the poly(A)-binding protein 2 (PABP2; PF3D7_0923900) and serine/arginine splicing factor 1 (SR1; PF3D7_0517300) were significantly enriched in the nonmethylated RNA oligonucleotide pulldown (Fig. 1B; Table S1).

10.1128/mBio.00661-21.4 TABLE S1 Results of the TMT-based quantification of proteins pulled down *in vitro* with m^6^A or A RNA oligonucleotides. Download Table S1, XLSX file, 0.03 MB. Copyright © 2021 Sinha et al. 2021 Sinha et al. https://creativecommons.org/licenses/by/4.0/ This content is distributed under the terms of the Creative Commons Attribution 4.0 International license.

Besides PfYTH.1, P. falciparum harbors a second YTH domain protein, called PfYTH.2 (PF3D7_0309800). Evolutionarily, both PfYTH.1 and PfYTH.2 are similar to YTH domain family (YTHDF) proteins found in humans and ECT2 in *Arabidopsis* (Fig. S1C). PfYTH.1 has a length similar to that of human YTHDF and includes a C-terminal YTH domain and an N-terminal zinc finger domain with an intermediate intrinsically disordered region (Fig. S1D). In contrast, PfYTH.2 has an N-terminal YTH domain, is half the length of PfYTH.1, and, in general, shows little homology to other eukaryotic YTH proteins. Yet, key residues of the YTH domain involved in m^6^A binding in other eukaryotes are conserved in both PfYTH.1 and PfYTH.2 (Fig. S1E). Given the higher potential of selective drug development with more divergent reader proteins, we next aimed to also identify PfYTH.2 as an m^6^A-binding protein in P. falciparum. First, an N-terminally glutathione *S*-transferase (GST)-tagged recombinant full-length PfYTH.2 protein was produced in Escherichia coli (Fig. 1C; Fig. S1F). In a dot blot assay using the same synthetic RNA oligonucleotides as for the *de novo* identification of m^6^A readers (Fig. S1B), recombinant PfYTH.2 bound to the methylated oligonucleotide in an RNA concentration-dependent manner. In contrast, recombinant PfYTH.2 did not bind to a nonmethylated control RNA oligonucleotide (Fig. S1B) of identical sequence and length (Fig. 1D). In contrast, two individual mutations (W46L and D143E) of conserved residues in PfYTH.2 known to facilitate m^6^A binding (16, 17) (Fig. S1E) led to decreased binding of the mutated PfYTH.2 to m^6^A (Fig. S1G), also indicating that the binding signal is dependent on the PfYTH.2 protein and not possible impurities or the GST tag of the recombinant protein.

To assess whether PfYTH.2 binds m^6^A in native P. falciparum mRNA, we next performed an *in vitro* protein-mRNA pulldown (Fig. S1H). Recombinant PfYTH.2 was immobilized on anti-GST magnetic beads and incubated with poly(A)-RNA purified from parasites at 36 hpi. Levels of m^6^A and N^7^-methylguanosine (m^7^G) in the input, supernatant, and immunoprecipitate were measured by liquid chromatography coupled to triple quadrupole mass spectrometry (LC-MS/MS). We observed a significant enrichment of m^6^A in the PfYTH.2-bound fraction after normalization with the corresponding unmodified nucleoside (rA) (Fig. 1E). Conversely, m^6^A/rA levels were substantially depleted in the supernatant compared to levels in the input (Fig. 1E). This trend was not observed for m^7^G in these same samples, suggesting that PfYTH.2 preferentially binds to m^6^A (Fig. S1I).

### PfYTH.2 associates with proteins involved in mRNA translation.

**FIG 2:**
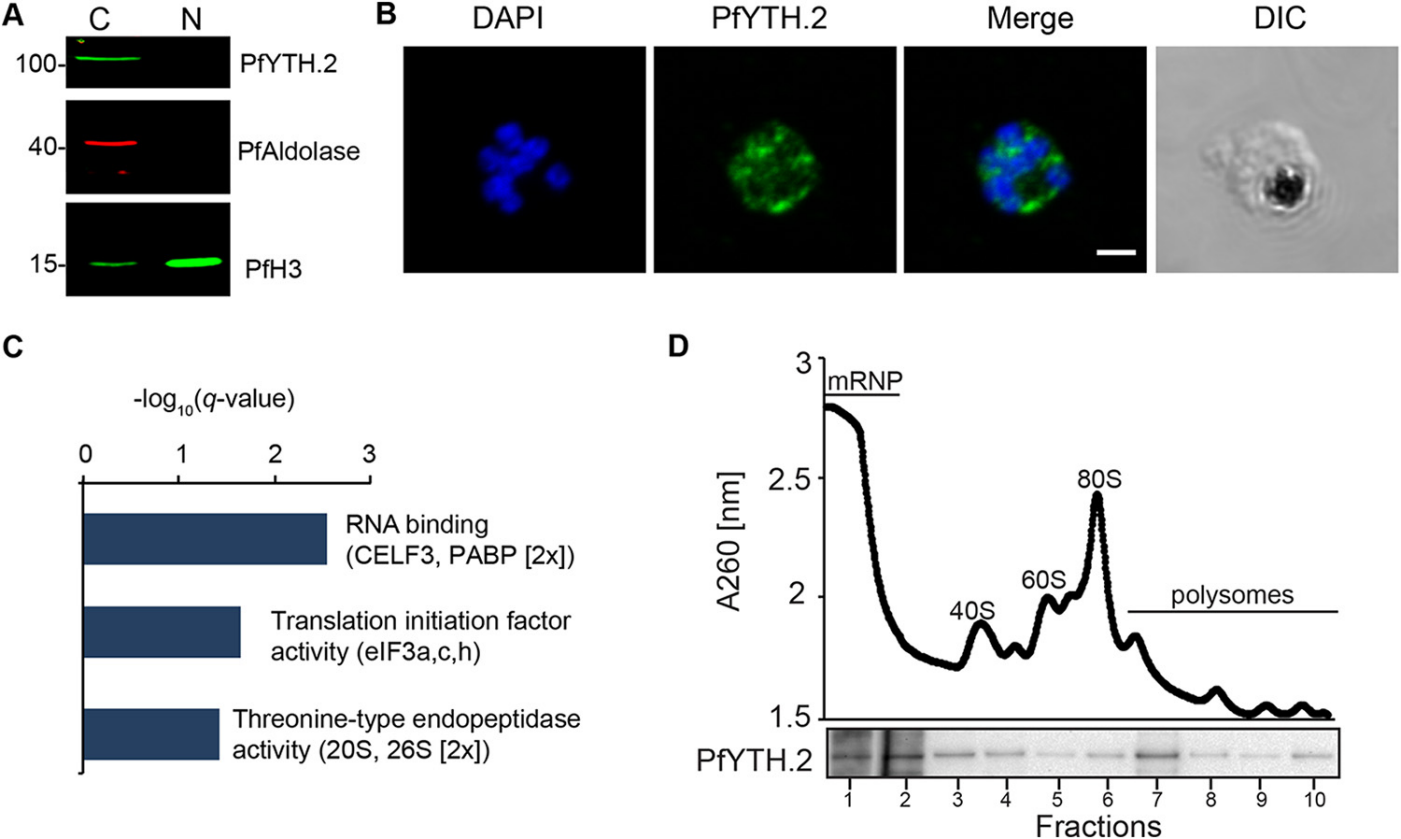
PfYTH.2 associates with the translational machinery. (A) Western blot analysis using anti-GFP antibodies to visualize PfYTH.2-sandwich (expected size, ∼116 kDa) shows the expression of the fusion protein and its localization to the cytoplasm. Histone H3 (PfH3) and aldolase (PfAldolase) serve as markers for the cytoplasmic (C) and nuclear (N) fractions. Numbers on the left indicate protein size in kilodaltons. (B) Immunofluorescence assay using anti-GFP antibodies shows the main localization of PfYTH.2 (green) to the parasite cytoplasm. Nucleus stained with Hoechst (blue). Bar, 2 μm. (C) Gene ontology (GO) enrichment analysis of proteins that were co-immunoprecipitated specifically with PfYTH.2 and identified by mass spectrometry (see Table S2). (D) Absorbance (260 nm) of a sucrose gradient of PfYTH.2-sandwich cell lysates (top) showing different cell fractions containing free mRNA ribonucleoprotein complexes (mRNP), small and large ribosome subunits (40S and 60S), monosomes (80S), and mRNAs associated with multiple ribosomes (polysomes). (Bottom) Western Blot using anti-GFP antibodies identifies PfYTH.2 to be present in the mRNP, monosome, and polysome fractions corresponding to the profile above.

To investigate the function of PfYTH.2 *in vivo*, we added a green fluorescent protein (GFP) tag flanked on either side by two FK506 binding protein (FKBP) domains to the C terminus of the endogenous copy of PfYTH.2 (PfYTH.2-sandwich) (see Fig. S2A) using the selection-linked integration approach as in reference 18. Integration of the construct was confirmed by PCR (Fig. S2B) and sequencing, and a Western blot analysis showed the PfYTH.2 fusion protein at the expected size predominantly in the cytoplasm of trophozoites (Fig. 2A). An immunofluorescence assay (IFA) confirmed this localization of the PfYTH.2 fusion protein primarily to the cytoplasm as well (Fig. 2B).

10.1128/mBio.00661-21.2 FIG S2 (A) Schematic of the PfYTH.2 tagging strategy. The C-terminal tag was integrated into the genomic PfYTH.2 locus by selection-linked integration. hDHFR, human dihydrofolate reductase; FKBP, FK506 binding protein; GFP, green fluorescent protein; 2A, T2A skip peptide; Neo-R, neomycin resistance gene; asterisk, stop codon. Black arrow denotes direction of transcription. Red shapes, location of primers for PCR results shown in panel B. (B) PCR confirmation of integration of the 2×FKBP-GFP-2×FKBP (sandwich) tag into the PfYTH.2 locus. Location of the primer pairs are shown in panel A. Numbers on the left denote size in base pairs. (C) Schematic of the pLyn-FRB-mCherry plasmid (mislocalizer [ML]) that was transfected into PfYTH.2-sandwich parasites to generate the PfYTH.2-sandwich^ML^ strain. BSD, blasticidin s deaminase; FRB, FKBP-rapamycin binding domain; Lyn, plasma-membrane targeting peptide sequence (MGCIKSKGKDSAGA) (18, 52); nmd3, promoter of the 60S ribosomal export protein NMD3. Arrow denotes direction of transcription. (D) Venn diagram showing the distribution and overlaps of proteins that were co-immunoprecipitated with PfYTH.2 (sample [blue], ≥1 unique peptide in ≥1 replicate) that were present in the control sample (control [yellow], ≥1 unique peptide in ≥1 replicate) and those remained after filtering (filter [green], ≥2 unique peptide in ≥2 replicates and not present in the control). The specific proteins are highlighted in red (PfYTH.2 plus 12 putative interacting proteins). Download FIG S2, PDF file, 0.9 MB. Copyright © 2021 Sinha et al. 2021 Sinha et al. https://creativecommons.org/licenses/by/4.0/ This content is distributed under the terms of the Creative Commons Attribution 4.0 International license.

10.1128/mBio.00661-21.5 TABLE S2 Results of the PfYTH.2-sandwich protein co-immunoprecipitation. Download Table S2, XLSX file, 0.05 MB. Copyright © 2021 Sinha et al. 2021 Sinha et al. https://creativecommons.org/licenses/by/4.0/ This content is distributed under the terms of the Creative Commons Attribution 4.0 International license.

To identify proteins that interact with PfYTH.2, we performed protein co-immunoprecipitation with an anti-GFP antibody for trophozoite parasites followed by LC-MS. We identified 12 specific proteins (present with ≥2 unique peptides in at least 2 of 3 PfYTH.2-sandwich replicates and not present in the control) (Fig. S2D), including multiple proteins putatively associated with the translation machinery: three subunits of the eukaryotic initiation factor 3 (eIF3a, -c, and -h), two polyadenylate binding-like proteins (PABPs), and three proteosomal proteins linked to protein degradation. A third PABP (PABP1; PF3D7_1224300) was also found to be substantially more enriched but not uniquely present in the PfYTH.2 co-immunoprecipitation compared to that in the control GFP immunoprecipitation in wild-type (WT) parasites. In addition, we found gamete antigen 27 (PF3D7_1302100) and a CELF3-like protein (PF3D7_0823200) to specifically associate with PfYTH.2, among others (see Table S2). A gene ontology (GO) enrichment analysis of the putative PfYTH.2 interacting proteins showed a significant enrichment of molecular functions “RNA binding”, “translation initiation factor activity”, and “threonine-type endopeptidase activity” (Fig. 2C). In contrast, PfYTH.2 was not found to interact with PfYTH.1, further suggesting that the two proteins have independent mode of actions.

To further reveal a possible association of PfYTH.2 with either mRNA ribonucleoprotein complexes (mRNP) or the translation machinery, we fractionated non-cross-linked, early trophozoite PfYTH.2-sandwich cell extracts on a sucrose gradient. Western Blot analysis of the different fractions showed PfYTH.2 to be present in the mRNP, monosome, and polysome fractions (Fig. 2D). Together with the interaction of PfYTH.2 with other RNA-binding proteins and translational regulators, these data suggest that PfYTH.2 can associate with free and ribosome-associated mRNAs to possibly modulate mRNA homeostasis and/or translation.

### PfYTH.2 is essential for blood-stage development.

**FIG 3:**
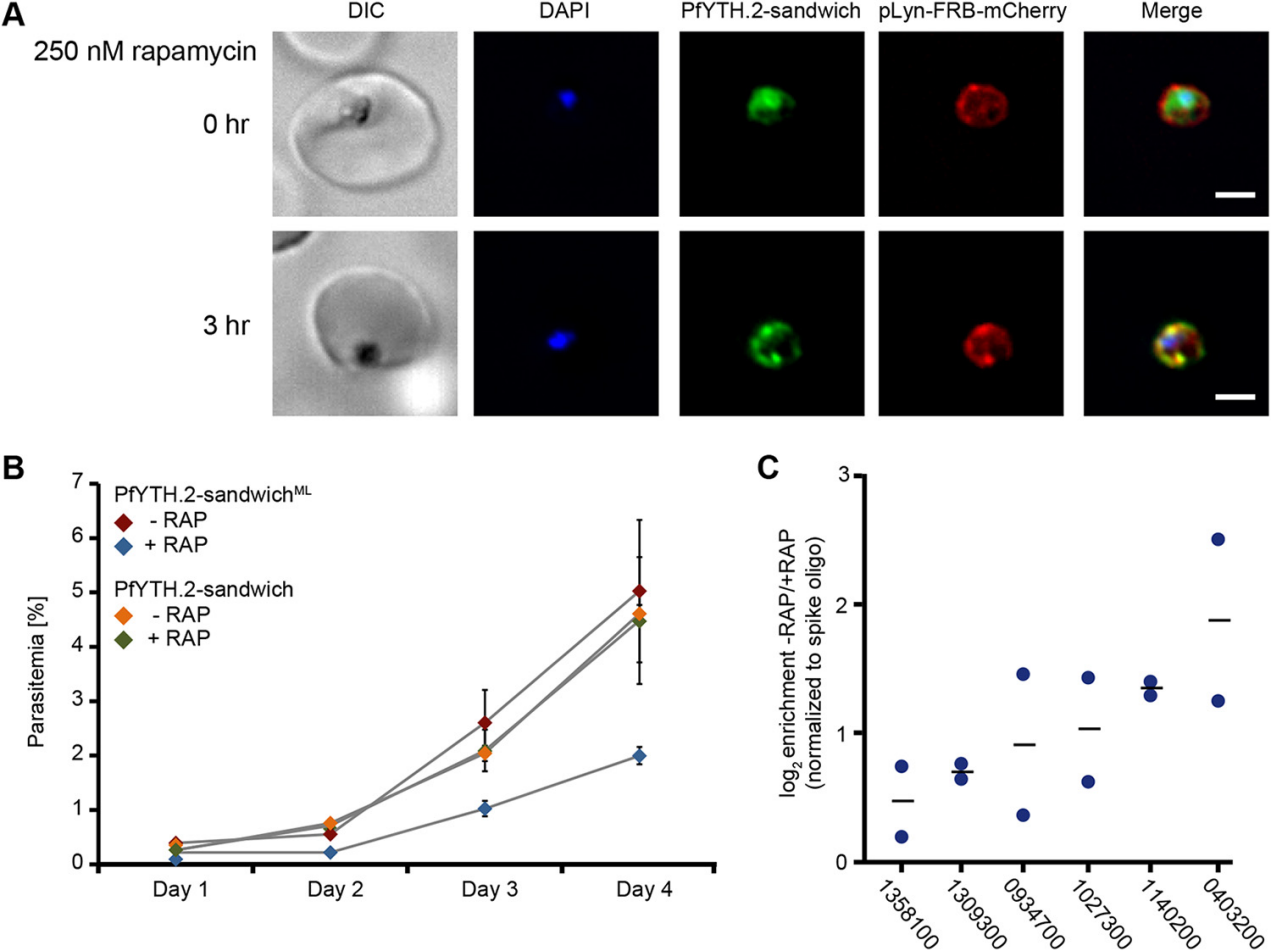
PfYTH.2 is essential for blood-stage asexual/replicative cycle. (A) Live-cell imaging of PfYTH.2-sandwich mislocalization following rapamycin addition in PfYTH.2-sandwich^ML^ parasites. Bar, 2 μm. (B) Growth curve over 4 days of PfYTH.2-sandwich and PfYTH.2-sandwich^ML^ parasites in the presence (250 nM final concentration, +RAP) or absence (−RAP) of rapamycin. Error bars represent standard deviations from six independent experiments. (C) Enrichment (log_2_) of PfYTH.2-bound transcripts in nontreated (−RAP) compared to that in knock-sideways (+RAP) PfYTH.2-sandwich^ML^ cells following PfYTH.2-RNA co-immunoprecipitation. Transcript levels were quantified by RT-qPCR. Blue circles represent log_2_ enrichment values from two independent biological replicates. Black lines represent the means. 0403200, putative pre-mRNA splicing factor; 0934700, putative UBX domain-containing protein; 1027300, peroxiredoxin; 1140200, conserved *Plasmodium* protein, unknown function; 1309300, putative U4/U6 small nuclear ribonucleoprotein PRP3; 1358100, putative Sas10 domain-containing protein.

Since PfYTH.2 was not mutable in a genome-wide mutagenesis screen (19), we generated a strain in which we could perform ligand-inducible relocalization (18) (i.e., knock sideways) of PfYTH.2 to determine the role of this protein in the IDC. For this, a plasmid expressing an FKBP-rapamycin-binding (FRB) domain fused to a plasma membrane-anchored peptide (Lyn) and mCherry (i.e., the “mislocalizer”) was transfected into the PfYTH.2-sandwich strain described above (Fig. S2C). Live-cell microscopy of the resulting strain (PfYTH.2-sandwich^ML^) showed localization of the Lyn-FRB-mCherry mislocalizer to the plasma membrane (Fig. 3A, top). Addition of the small-molecule ligand rapamycin to the cell culture, which induces dimerization of FKBP and FRB, resulted in the rapid relocalization of PfYTH.2-sandwich to the plasma membrane within 3 h (Fig. 3A, bottom).

To determine if PfYTH.2 knock-sideways leads to a functional knockdown, we compared growth rates with or without rapamycin between the PfYTH.2-sandwich and PfYTH.2-sandwich^ML^ strains. The PfYTH.2-sandwich and PfYTH.2-sandwich^ML^ strains grew at similar rates in the absence of rapamycin, and addition of rapamycin did not affect growth rates of PfYTH.2-sandwich parasites, indicating that these concentrations of rapamycin do not restrict parasite growth (Fig. 3B). However, PfYTH.2-sandwich^ML^ parasites grown in the presence of rapamycin showed a significantly decreased growth rate (Fig. 3B). This indicates that neither modification of the PfYTH.2 with the sandwich fusion nor presence of the mislocalizer affects cell growth, but that the inducible mislocalization of PfYTH.2 affects parasite replication and/or survival, presumably due to a knockdown of PfYTH.2 function.

To assess whether this mislocalization leads to a dissociation of PfYTH.2 and its putative target transcripts, we performed an *in vivo* PfYTH.2 RNA co-immunoprecipitation followed by reverse transcription-quantitative PCR (RIP-qPCR) on formaldehyde cross-linked PfYTH.2-sandwich^ML^ cells that were either treated with rapamycin for 3 h (+RAP) or nontreated (−RAP). All putative target transcripts investigated (i.e., previously identified to have an m^6^A peak [12]) were found to be more abundant under the nontreated condition than under the rapamycin-treated condition (Fig. 3C). This suggests that correct localization of PfYTH.2 within the cell is critical for its function and that its mislocalization does not lead to the relocation of the protein together with its bound transcript but induces the dissociation of the m^6^A reader from its target mRNA and mRNP complex.

### PfYTH.2 knockdown leads to an increase in translational efficiency.

**FIG 4:**
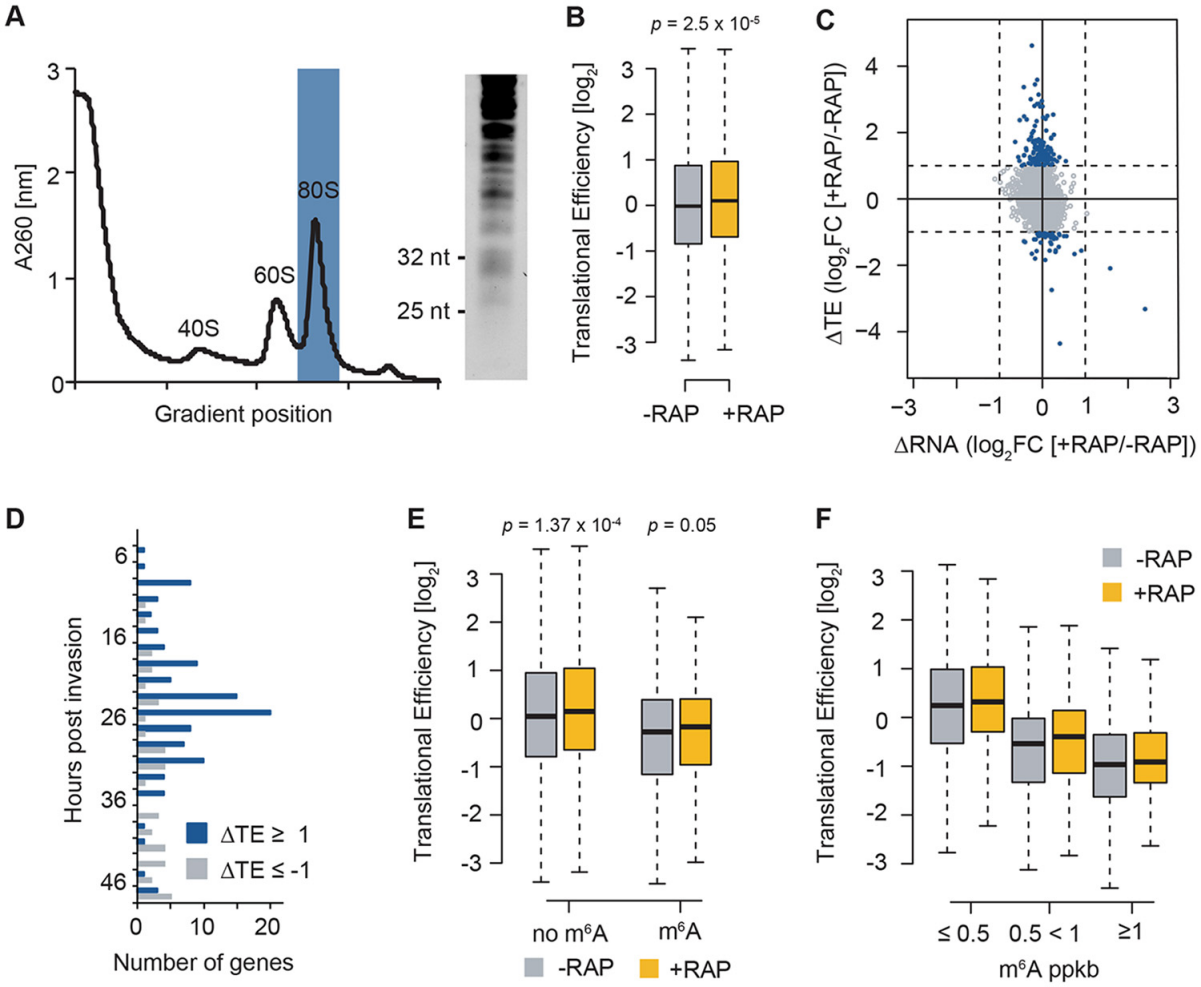
Ribo-seq identifies PfYTH.2 as a modulator of translational efficiency in P. falciparum. (A) Absorbance profile of the sucrose gradient (measured at 260 nm) of the RNA harvested from parasites in the absence of rapamycin after RNase I treatment. The 80S monosome fraction (blue) was collected, and the small RNA-containing fraction ranging from 26 to 32 nt was size selected on a 15% TBE-urea gel to enrich for ribosome-protected fragments (right). (B) Box plot showing translational efficiencies (calculated as fragments per kilobase of exon per one million mapped reads [FPKM] RPF/FPKM mRNA) in PfYTH.2-sandwich^ML^ parasites after 3 h incubation without (−RAP, gray) or with 250 nM rapamycin (+RAP, yellow). Center line, median; box limits, first and third quartiles; whiskers, 1.5× interquartile range. *P* values were calculated using a two-sided Mann-Whitney *U* test. *n *= 3,745. (C) Scatterplot showing the log_2_ fold change in translational efficiency (ordinate, ΔTE) and total mRNA abundance (abscissa, ΔRNA) between PfYTH.2-sandwich^ML^ parasites after 3 h incubation with (+RAP) or without (−RAP) rapamycin. Blue, transcripts with a log_2_FC TE of ≥1 or ≤−1. (D) Histogram showing the hours postinvasion of the RBCs at which genes with a log_2_FC in TE of ≥1 (blue) or ≤−1 (gray) are maximally transcribed. Transcription data set derived from reference 9. (E) Box plot of translational efficiencies for transcripts without (no m^6^A) or with an m^6^A peak (m^6^A) measured in PfYTH.2-sandwich^ML^ parasites after 3 h incubation without (−RAP, gray) or with (+RAP, yellow) rapamycin. Center line, median; box limits, first and third quartiles; whiskers, 1.5× interquartile range. *P* values were calculated using a two-sided Mann-Whitney *U* test. No m^6^A, *n* = 3,266; m^6^A, *n* = 481 (data set derived from reference 12). (F) Box plot of translational efficiencies measured in PfYTH.2-sandwich^ML^ parasites after 3 h incubation without −RAP, gray) or with (+RAP, yellow) rapamycin for m^6^A-methylated transcripts as a function of m^6^A peak density (m^6^A peaks per kilobase of exon [ppkb]). Center line, median; box limits, first and third quartiles; whiskers, 1.5× interquartile range. m^6^A ppkb of ≤0.5, *n* = 388; m^6^A ppkb of 0.5 to <1, *n* = 144; m^6^A ppkb of ≥1, *n* = 72 (data set derived from reference 12).

Given the association of PfYTH.2 with multiple components of the translational machinery, we next assessed the effect of PfYTH.2 knock-sideways on translational efficiencies. Synchronized PfYTH.2-sandwich^ML^ parasites were incubated for 3 h with (+RAP) or without (−RAP) rapamycin, and RNA was harvested at 24 hpi. We performed ribosome profiling by RNase treatment of the protein lysates to digest actively translating polysomes into individual monosomes that remain associated with the mRNA fragment they were bound to at the time point of sampling (i.e., the ribosome-protected fragments [RPF] shielded from the nuclease). The 80S monosome fraction of the digested lysate was purified from a 10% to 50% sucrose gradient (Fig. 4A, left) followed by gel size selection of the 25- to 32-nucleotide (nt) RPF fraction (Fig. 4A, right). These RPFs were sequenced in parallel with mRNA from the same samples, allowing us to calculate the density (i.e., coverage) of RPFs that correspond to the number of actively translating ribosomes. Normalized to the relative abundance of the mRNA transcript obtained from the mRNA sequencing (mRNA-seq) performed in parallel, this provides a relative measure of translational efficiencies for each transcript (TE) (see Table S3) (20). We observed an overall positive correlation with two previously published P. falciparum TE data sets (10, 21), despite differing sampling time points, RPF preparation protocols, and TE calculation formulas (21), suggesting that our method was robust (see Fig. S3A).

10.1128/mBio.00661-21.3 FIG S3 (A) Comparison of translational efficiencies of PFYTH.2-sandwich^ML^ parasites (cultured without rapamycin [−RAP]) and TE values measured in previous studies. TE reported by Caro et al. (10) was measured at 31 hpi following parasite incubation with cycloheximide. TE reported by Ng et al. (21) was measured from quantitative proteomics and mRNA sequencing at 24 hpi. *r*, Pearson's product moment correlation coefficient. Black line, linear regression line with 95% confidence region (grey area). (B) Box plot of translational efficiency for each replicate in PfYTH.2-sandwich^ML^ parasites grown with (+RAP, yellow) and without (−RAP, grey) rapamycin. *n *= 3,745. (C) Log_2_ fold changes of translational efficiencies (ΔTE) as a function of m^6^A density. ppkb, m^6^A peaks per kilobase of exon. Blue dots and lines represent the means and ± one standard deviation, respectively. m^6^A ppkb of ≤0.5, *n* = 388; m^6^A ppkb of 0.5 to <1, *n* = 144; m^6^A ppkb of ≥1, *n* = 72. (D) Schematic of domain structure of IGF2BP1, FMRP, and PfKH.1 full-length proteins. RRM, RNA recognition motif; KH, K homology domain; FXMR, Fragile X mental retardation. (E) Protein alignment of the two KH repeat domains in human IGF2BP1 and PfKH.1. Download FIG S3, PDF file, 1.2 MB. Copyright © 2021 Sinha et al. 2021 Sinha et al. https://creativecommons.org/licenses/by/4.0/ This content is distributed under the terms of the Creative Commons Attribution 4.0 International license.

10.1128/mBio.00661-21.6 TABLE S3 FPKM-derived translational efficiencies and changes in TE and RNA abundance in PfYTH.2-sandwich^ML^ parasites grown with or without rapamycin. Download Table S3, XLSX file, 0.8 MB. Copyright © 2021 Sinha et al. 2021 Sinha et al. https://creativecommons.org/licenses/by/4.0/ This content is distributed under the terms of the Creative Commons Attribution 4.0 International license.

A comparison of translational efficiencies for all genes showed a significant overall increase in TE following knock-sideways of PfYTH.2 (Fig. 4B; Fig. S3B). A gene-specific calculation of TE revealed a similar pattern, with significantly more transcripts showing an increase (log_2_ fold change [FC] [ΔTE] ≥ 1, *n *= 128) compared to a decrease (log_2_FC [ΔTE] ≤ −1, *n *= 45) in TE (two-tailed χ^2^, *P* < 0.0001) (Fig. 4C; Table S3). In contrast to the changes in TE (ΔTE), changes in mRNA abundance (ΔRNA) are less pronounced, suggesting that PfYTH.2 knock-sideways primarily affects ribosome occupancy rather than underlying mRNA abundance (Fig. 4C). Most transcripts with increased TE (log_2_FC ≥ 1) reach peak transcription at 24 to 26 hpi (i.e., the time point of sample collection) (9), whereas a similar pattern is not observed for transcripts with decreased TE (log_2_FC ≤ −1) (Fig. 4D). Surprisingly, following knock sideways of PfYTH.2, TE significantly increases for transcripts with and without identified m^6^A peaks (12), which might be due to an incomplete annotation of m^6^A sites which was performed using an anti-m^6^A antibody-based approach (12) (Fig. 4E). A further classification of m^6^A-methylated transcripts based on m^6^A peak density revealed an inverse correlation between TE and m^6^A density (Fig. 4F) in the untreated parasites. Following PfYTH.2 knock-sideways, TE increases independently of m^6^A density (Fig. 4F); however, transcripts with higher m^6^A density show slightly higher average changes in TE (Fig. S3C). Altogether, the increase in translational efficiencies following inducible PfYTH.2 knock-sideways suggests that this protein is an m^6^A-sensitive modulator of translation with a putatively repressive function.

## DISCUSSION

In this study, we identify two YTH proteins as bona fide m^6^A-binding factors in the human malaria parasite P. falciparum. Characterization of the divergent PfYTH.2 showed that it interacts with components of the translational machinery and serves a repressive function with regard to translation. The YTH domain has been identified as an evolutionarily conserved m^6^A-binding moiety found in a range of m^6^A reader proteins in model eukaryotes, including yeast, fly, *Arabidopsis*, and mammal. Importantly, while certain YTH domain proteins in mammalian cells are functionally redundant (13), the overall sequence divergence and the distinct expression profiles of the two P. falciparum YTH proteins suggest that they have nonredundant functions. In line with the conservation of key residues within the YTH domain, which are involved in RNA and m^6^A binding in other eukaryotes, we demonstrated that PfYTH.2 is an m^6^A-binding protein with dot blot assay and RNA pulldown followed by LC-MS/MS. While our attempt to identify other m^6^A readers showed binding for PfYTH.1, the lack of PfYTH.2 in the oligonucleotide pulldown experiment might be either due to suboptimal ionization during mass spectrometry or due to its short length, which could require a more sensitive mass spectrometry analysis to identify the fewer corresponding peptides coming from this protein. Indeed, we note that PfYTH.2 is sparsely detected in other P. falciparum proteomics experiments and does not feature high peptide counts even in the targeted PfYTH.2 protein co-immunoprecipitation (see Table S2 in the supplemental material). In addition, it is also possible that despite similar mRNA expression levels, PfYTH.1 is the predominant m^6^A-binding protein at this stage of parasite development. Nevertheless, the m^6^A oligonucleotide pulldown approach also identified other putative m^6^A reader proteins, among which is the K homology domain-containing protein PfKH.1. Indeed, the KH domain-containing protein IGF2BP1 was recently characterized as a putative m^6^A reader in mammalian cells (22, 23). Both IGF2BP1 and PfKH.1 share a similar domain architecture that includes two highly conserved tandem KH domain repeats (Fig. S3D and E). In addition, several other KH domain-containing proteins, including the fragile X mental retardation protein (FMRP), have been identified in mammalian m^6^A interactome screens as putative m^6^A readers (24, 25). However, while it is possible that the KH domain is an evolutionarily conserved m^6^A-binding moiety, it is similarly possible that PfKH.1 is a PfYTH.1-interacting protein, as recently reported in Drosophila melanogaster (26), and thus appears as enriched in the oligonucleotide pulldown experiment.

PfYTH.2 was not mutable in a PiggyPac mutagenesis screen (19), suggesting that this gene is essential for parasite survival, similar to other components of the m^6^A pathway (12). Thus, we used an inducible knock-sideways approach to functionally knock down PfYTH.2, leading to the inhibition of parasite growth likely due to the dissociation of PfYTH.2 from its target transcripts, as evidenced by our RIP-qPCR data. The growth deficiency following PfYTH.2 further suggest that its function cannot be complemented by PfYTH.1, which is also highlighted by the negligible changes in gene expression of PfYTH.1 (log_2_ fold change, −0.06) or PfYTH.2 (log_2_ fold change, 0.04) un the PfYTH.2 knock-sideways condition (Table S3). Overall, this provides further evidence for the divergent functions of these two proteins.

A knockdown with a duration of only 3 h and subsequent comparative ribosome profiling revealed that PfYTH.2 likely acts as a repressor of mRNA translation, especially for high-abundance transcripts. These data suggest that PfYTH.2-mediated modulation of translation could act as a counterbalance to fine-tune translation of an otherwise more rigid transcriptional program. Importantly, an increase in translational efficiency was seen for mRNA transcripts with or without an m^6^A peak (as identified by m^6^A-seq [12]) following PfYTH.2 knock-sideways. Although our dot blot and LC-MS/MS analyses showed a clear preference of PfYTH.2 for m^6^A-modified transcripts, it is possible that PfYTH.2 might also act on nonmethylated mRNA transcripts. On the other hand, it is more likely that a substantial fraction of the transcripts categorized here as “nonmethylated” do actually contain m^6^A, as the number of identified m^6^A peaks is an underestimate of the true extent of m^6^A transcript modification in P. falciparum. Indeed, our previous quantitative LC-MS/MS measurements of global m^6^A levels suggested that there are up to 10 times more m^6^A residues than m^6^A peaks that were annotated using an antibody-based approach (12). Thus, many of the transcripts categorized as nonmethylated could in fact also contain m^6^A methylation sites.

The protein co-immunoprecipitation of PfYTH.2 identified multiple proteins that provide possible insight into the putative function of PfYTH.2 but for which functional studies are largely missing. Among those are several polyadenylate binding proteins (PABPs), which are well annotated and conserved in *Plasmodium* (27) and which are known regulators of mRNA translation in other eukaryotes (28). While the exact molecular function of other interacting proteins such as the CELF3-like protein or gamete antigen 27 in P. falciparum remains unknown, we also identified several components of the proteasome, which in a recent protein interaction screen were found to closely associate with the eIF3 complex in P. falciparum (29). The interaction of PfYTH.2 with multiple components of the eIF3 complex, in combination with our ribosome profiling (Ribo-seq) data and the presence of PfYTH.2 in in the mRNP but also the monosome and polysome fractions, strongly suggests that this m^6^A reader can act as a modulator of mRNA translation.

The question remains as to how PfYTH.2 might repress translation. The direct interaction of PfYTH.2 with PABPs and multiple eIF3 subunits but not with other proteins of the translation initiation complex (e.g., eIF4) or ribosomal proteins could suggest that PfYTH.2 impairs assembly of the intact translational machinery. Translational repression via interference with the translation initiation machinery has been observed in multiple model systems and can be mediated by microRNAs (miRNAs) (30), the UNR/Sxl complex in D. melanogaster (31), or the eIF4 binding protein 1 (32). On the other hand, the presence of PfYTH.2 in polysome fractions could also point toward a steric inhibition of ribosomes during translation elongation. Finally, cytoplasmic PfYTH.2 could also sequester m^6^A-methylated transcripts away from the translational machinery, leading to translational repression. Indeed, m^6^A-mediated mRNA sequestration by RNA-binding proteins and formation of biomolecular condensates was recently shown to coincide with translational repression (33). In addition, eIF3, PABP1, and the YTH-domain family protein 2 are known components of biomolecular condensates such as stress granules in mammalian cells (33). Importantly, PABP1 also colocalizes with translationally repressed biomolecular condensates in the host-to-vector transmission stage (i.e., gametocytes) and interacts with other translational repressors in *Plasmodium* (34, 35).

Widespread translational repression has been observed to the highest extent in gametocytes and sporozoites (i.e., the vector-to-host transmission stage) (36, 37). As PfYTH.2 is most highly expressed in these stages (Fig. S1A), it is possible that this protein is also involved in the regulation of translation in these transmission stages. What remains unclear, however, is whether PfYTH.1 and PfYTH.2 specifically target different cohorts of mRNA transcripts or m^6^A methylation sites, especially at life cycle stages such as the IDC, when these proteins show similar expression patters.

In conclusion, here we identify two YTH domain proteins as m^6^A-binding factors in P. falciparum. The identification of these proteins will be instrumental in further understanding how the prevalent m^6^A modification of the transcriptome in this unicellular pathogen is translated into distinct biological outcomes across the parasite life cycle.

## MATERIALS AND METHODS

### Parasite culture.

Asexual blood-stage P. falciparum parasites (strain 3D7) were cultured as described previously (15). Briefly, parasites were cultured in human red blood cells (RBCs) in RPMI 1640 culture medium (Thermo Fisher 22400097) supplemented with 10% (vol/vol) AlbuMAX I (Thermo Fisher 11020039), hypoxanthine (0.1 mM final concentration; CC-Pro Z-41-M), and 10 mg gentamicin (Sigma G1397-10ML) at 4% hematocrit and under 5% O_2_, 3% CO_2_, at 37°C. Red blood cells were obtained from the Etablissement Français du Sang with approval number HS 2016-24803. Parasite development was monitored by Giemsa staining.

For sampling of highly synchronous parasites during the IDC, the synchronous schizonts were enriched by plasmagel flotation shortly before reinvasion, followed by a sorbitol lysis 6 h later. The 0-h time point was considered to be 3 h after plasmagel flotation. Parasites were collected at 4% hematocrit and ∼2% to 3% parasitemia.

### Parasite growth assay.

To measure parasite growth kinetics, PfYTH.2-sandwich and PfYTH.2-sandwich^ML^ parasites were tightly synchronized and grown either in the presence or absence of 250 nM rapamycin (Sigma R8781). The growth curve was replicated in three distinct batches of RBCs on a 96-well plate in 200 μl complete culture medium per well (and 2 μg/ml blasticidin for the PfYTH.2-sandwich^ML^ replicates). Each day, 5 μl of cell culture was collected and fixed in 45 μl 0.025% glutaraldehyde–phosphate-buffered saline (PBS) for 60 min at 4°C. To quench the reaction, parasites were spun down (5 min, 3,250 × *g*, 4°C), the supernatant was removed, and cells were suspended in 200 μl 15 mM NH_4_Cl-PBS. Parasite nuclei were stained using SYBR green (Sigma S9430) for 30 min and (non)infected RBCs were counted on a CytoFlexS flow cytometer (Beckman Coulter).

### Recombinant expression of PfYTH.2.

The full-length coding sequence of the PfYTH.2 gene (PF3D7_0309800) was PCR amplified from cDNA to remove introns and cloned into a pGEX expression vector. The recombinant protein carrying an N-terminal GST tag was made in BL21(DE3) bacteria. The bacterial pellet was lysed (50 mM Tris, 50 mM NaCl, 5 mM EDTA with protease inhibitor [Sigma 4693159001]), sonicated, and centrifuged (18,000 × *g* for 60 min at 4°C) to obtain the soluble fraction, which was allowed to bind with glutathione Sepharose beads (GE Healthcare) at 4°C. The beads were washed with 5 mM EDTA-PBS (pH 7.4) followed by ATP buffer (50 mM Tris, 2 mM ATP, 10 mM MgSO_4_, pH 7.4), and the recombinant protein was then eluted with elution buffer (50 mM Tris, 10 mM reduced glutathione, pH 8). Two PfYTH.2 mutants (W46L and D143E) were generated by PCR (PfuUltra; Agilent) using mutagenesis primers (see Table S4 in the supplemental material) and the pGEX-PfYTH.2 plasmid as the template. Recombinant protein expression and purification were performed as described above. Purified recombinant proteins were migrated on gel (Mini-Protean TGX stain-free gel; Bio-Rad) and transferred to membrane (Trans-Blot Turbo; Bio-Rad). Western blotting was performed by overnight incubation with anti-GST antibody (GE Healthcare 27457701) at 4°C with gentle agitation. The membrane was rinsed three times with PBS with Tween 20 (PBST) and then incubated with horseradish peroxidase (HRP)-coupled secondary antibody (donkey anti-goat IgG-HRP; Santa Cruz sc-2020). The membrane was rinsed as described above and revealed by chemiluminescence (Thermo Fisher 34580). Gels and membranes were imaged with a ChemiDoc XRS+ (Bio-Rad).

10.1128/mBio.00661-21.7 TABLE S4 Primers used in this study. Download Table S4, XLSX file, 0.01 MB. Copyright © 2021 Sinha et al. 2021 Sinha et al. https://creativecommons.org/licenses/by/4.0/ This content is distributed under the terms of the Creative Commons Attribution 4.0 International license.

### Dot blot assay.

Positively charged nylon membranes (GE Healthcare RPN303B) were rinsed with ultrapure water, allowed to dry, and then spotted with three different concentrations (1 μg, 100 ng, and 10 ng) of m^6^A or A oligonucleotide (synthesized by GE Dharmacon) (Fig. S1B). The spots were allowed to dry before the RNA was UV cross-linked. Membranes were rinsed in PBST and then blocked with 5% low-fat milk in PBST for 1 h. Ten micrograms of purified recombinant protein was added and incubated for 2 h at room temperature with gentle agitation. Membranes were rinsed three times with PBST for 10 min each, followed by overnight incubation with an anti-GST antibody (GE Healthcare 27457701) at 4°C with gentle agitation. The membrane was rinsed three times with PBST and then incubated with HRP-coupled secondary antibody (donkey anti-goat IgG-HRP; Santa Cruz sc-2020). The membrane was rinsed as described above, developed by chemiluminescence (Thermo Fisher 34580), and imaged with a ChemiDoc XRS+ (Bio-Rad).

### *In vitro* RNA pulldown.

Total RNA from highly synchronous parasites was extracted from samples collected at 36 hpi. Briefly, red blood cells were lysed with 0.075% saponin, and the parasite cell pellet was washed once with Dulbecco’s PBS (DPBS) and then resuspended in 700 μl QIAzol (Qiagen 79306). Total RNA was extracted using the miRNeasy minikit (Qiagen 217004) according to the manufacturer’s instructions, followed by poly(A) RNA enrichment using the Dynabeads mRNA purification kit (Thermo Fisher 61006). Five hundred nanograms of mRNA was diluted to 100 μl and mixed with 100 μl of 2× IPP buffer (300 mM NaCl, 0.2% NP-40, 20 mM Tris [pH 7.4], 80 U/ml RNase inhibitor). Twenty microliters of mRNA at this concentration was saved as input sample for m^6^A analysis. For each sample, 50 μl GST-affinity magnetic beads (Thermo Fisher 88821) were washed 2× with 200 μl of IPP buffer.

Recombinant PfYTH.2 was concentrated (Vivaspin; GE Healthcare) and buffer exchanged into IPP buffer (150 mM NaCl, 0.1% NP-40, 10 mM Tris [pH 7.4], 40 U/ml RNase inhibitor). One hundred microliters of recombinant PfYTH.2 in IPP buffer at a concentration of 4.8 μM was then incubated with GST-affinity magnetic beads for 2 h at 4°C on a rotator. The PfYTH.2-GST-bound beads were then washed 3 times with 200 μl IPP buffer and incubated with 180 μl of mRNA for 4 h on a rotator at 4°C. The first unbound flowthrough was saved for analysis. The beads were then washed 4 times with 200 μl of IPP buffer, and QIAzol was added to the collected samples (input, flowthrough, and the PfYTH.2-bound mRNAs). The RNA was purified according to the manufacturer’s instructions using solid-phase extraction (Thermo Fisher K157001). The purified fraction was dissolved in 50 μl water.

### Protein-RNA pulldown and protein mass spectrometry.

Magnetic streptavidin beads (Thermo Fisher 65001) were washed and prepared as per the manufacturer’s instructions and resuspended in 2× BW buffer (10 mM Tris-HCl [pH 7.5], 1 mM EDTA, 2 M NaCl) at a final concentration of 5 μg/μl. An equal volume of RNA baits either with or without m^6^A were immobilized on the beads via a 5′ biotin tag (Fig. S1B). For each reaction mixture, 3.5 μg (400 pmol) of RNA oligonucleotides was incubated with 50 μl (125 μg) of beads for 15 min at room temperature using gentle rotation. The RNA-coated beads were separated with a magnet for 3 min and washed thrice with 1× BW buffer and once with lysis buffer (25 mM Tris-HCl [pH 7.5], 10 mM NaCl, 1% IGEPAL, 1.5 mM MgCl_2_, protease inhibitor). Trophozoite-stage parasites were separated from the host red blood cells by incubation with 0.075% saponin in PBS followed by centrifugation for 4 min at 2,400 × *g*. The pellet was washed twice with ice-cold PBS, resuspended in 10 volumes of lysis buffer, and incubated on ice for 30 min. This suspension was then transferred to a prechilled bouncer homogenizer and lysed with 200 strokes. The suspension was centrifuged at 13,000 × *g* at 4°C to pellet the insoluble fraction. One hundred microliters of the parasite lysate was applied to the beads for 4 h on a gentle rotator at 4°C. The beads were subsequently washed thrice with the lysis buffer followed by three washes of 25 mM ammonium bicarbonate. The beads were then reduced, alkylated, digested, and desalted. Protein peptides were subsequently labeled with TMT labels (Thermo Fisher 90110) as per the manufacturer’s instructions.

The protein mass spectrometry was performed as described previously (15). Briefly, peptides were separated by reverse-phase high-performance liquid chromatography (HPLC) (Thermo Easy nLC1000) using a commercial analytical column (Thermo Acclaim PepMax100) over a 90-min gradient before nanoelectrospray using an Orbitrap Fusion spectrometer (Thermo Scientific). The mass spectrometer was operated in a data-dependent mode. Full-scan MS parameters were as follows: resolution of 70,000 across 350 to 2,000 *m/z*, automatic gain control (AGC) of 3e^6^, and maximum injeciton time (IT) of 50 ms. The full MS scan was followed by MS/MS for the top 15 precursor ions in each cycle with a normalized collision energy (NCE) of 28 and dynamic exclusion of 30 s. Raw mass spectral data files (.raw) were searched using Proteome Discoverer (Thermo Fisher) and Mascot (version 2.4.1) (38). Mascot search parameters were as follows: 10 ppm mass tolerance for precursor ions, 15 mmu for fragment ion mass tolerance, 2 missed cleavages of trypsin, fixed modification was carbamidomethylation of cysteine, variable modifications were methionine oxidation, serine, threonine, and tyrosine phosphorylation, and cam-thioproponoyl. Only peptides with a Mascot score of greater than or equal to 25 and an isolation interference of less than or equal to 30 were included in the data analysis.

### Analysis of mRNA modifications by LC-MS/MS.

The input, flowthrough, and PfYTH.2-bound mRNA was hydrolyzed enzymatically as described previously (15) using the following components in the buffer mix: 10 mM Tris-HCl (pH 7.9), 1 mM MgCl_2_, 5 U Benzonase (Merck 71206), 50 μM desferrioxamine (Sigma D9533), 0.1 μg/μl pentostatin (Sigma SML0508), 100 μM butylated hydroxytoluene (Sigma W218405), 0.5 μg/μl tetrahydrouridine (Calbiochem 584222), 5 U bacterial alkaline phosphatase (Thermo Fischer 18011015), and 0.05 U phosphodiesterase I (Sigma P3243). Hypersil GOLD aQ column (100 mm by 2.1 mm, 1.9 μm; Thermo Scientific 25305) was used to resolve the digested ribonucleosides in a two-buffer eluent system (buffer A, 0.1% formic acid in water; buffer B, 0.1% formic acid in acetonitrile). HPLC was performed at a flow rate of 300 μl/min at 25°C. The gradient of 0.1% formic acid in acetonitrile was as follows: 0 to 12 min, held at 0%; 12 to 15.3 min, 0% to 1%; 15.3 to 18.7 min, 1% to 6%; 18.7 to 20 min, held at 6%; 20 to 24 min, 6% to 100%; 24 to 27.3 min, held at 100%; 27.3 to 28 min, 100% to 0%; 28 to 41 min, 0%. The HPLC column was directly connected to an Agilent 6490 triple quadrupole mass spectrometer with electrospray ionization (ESI) Jetstream ionization operated in positive-ion mode. The voltages and source gas parameters were as follows: gas temperature, 50°C; gas flow, 11 liters/min; nebulizer, 20 lb/in^2^; sheath gas temperature, 300°C; sheath gas flow, 12 liters/min; capillary voltage, 1,800 V; and nozzle voltage, 2,000 V. The molecular transition ions were quantified in multiple-reaction monitoring (MRM) mode as in reference 21.

### Identification and quantification of mRNA modifications by LC-MS/MS.

LC/MS data were extracted using the MassHunter qualitative and quantitative analysis software (version B06.00). To account for any background signal possibly contributing from the salts and enzymes in the digestion buffer, the signal intensity (i.e., area under the curve) for each ribonucleoside was first subtracted from a matrix sample (without RNA). To calculate relative levels of each ribonucleoside and to adjust for different injection amounts of RNA in each sample, the matrix-corrected intensity of the m^6^A was then divided by the intensity of the canonical ribonucleoside (i.e., rA).

### Generation of tagged PfYTH.2 cell lines and knock-sideways.

To generate a mislocalizable GFP-tagged version of PfYTH.2, we integrated two FK506 binding protein (FKBP) domains, followed by GFP, followed by two more FKBP domains to the 3′ end of the endogenous copy of the PfYTH.2 gene. This was followed by a 2A skip peptide and the neomycin resistance protein to allow integration of the whole cassette using the selection-linked integration approach described by Birnbaum and colleagues (18) (Fig. S2A). A 528-nt homology region at the 3′ end of PF3D7_0309800 was amplified using the primer pair PfYTH2-HR_F/R (Table S4) and cloned into the NotI and AvrII sites of the pSLI-sandwich (0807600) plasmid (Addgene plasmid 85790) described in reference 18. P. falciparum parasites (strain 3D7) were transfected and selected for integrants as previously described (18). Integration into the correct locus was verified using the primer pairs Locus_F/R, 5′arm_F/R, and 3′arm_F/R (Table S4).

To generate the PfYTH.2-sandwich^ML^ cell line, the PfYTH.2-sandwich parasites were subsequently transfected with a plasmid expressing the Lyn-mCherry plasma membrane mislocalizer (pLYN-FRB-mCherry-nmd3-BSD; Addgene plasmid 85796), and transfectants were selected as previously described (18).

### Protein fractionation and Western Blot analysis.

Trophozoite-stage parasites were separated from the host red blood cells by incubation with 0.15% saponin (Sigma) followed by centrifugation for 4 min at 2,200 × *g*. The parasite pellet was resuspended in 10 volumes of cytoplasmic extraction buffer (20 mM HEPES [pH 7.9], 10 mM KCl, 1 mM EDTA, 1 mM EGTA, 0.3% NP-40, 1 mM dithiothreitol [DTT], and protease inhibitors), incubated on ice for 5 min, and then centrifuged at 2,200 × *g* for 5 min at 4°C. The supernatant was collected as the cytoplasmic fraction, and the insoluble pellet was washed three times with the cytoplasmic extraction buffer. The insoluble pellet was incubated in 10 volumes of radioimmunoprecipitation assay (RIPA) buffer (Thermo Fisher) with protease inhibitors and 5 U Benzonase (Millipore). This fraction was then centrifuged at 13,000 × *g* for 10 min at 4°C to pellet down insoluble aggregates and hemazoin, and the supernatant was collected as the nuclear fraction. Both fractions were boiled at 95°C for 5 min after addition of 6× Laemmli loading dye. Proteins were separated in 12% SDS-polyacrylamide gels and transferred to low-fluorescence polyvinylidene difluoride (PVDF) membranes (Bio-Rad). The blots were blocked with Odyssey blocking buffer (PBS base) (LI-COR) for 1 h at room temperature. Primary antibodies against aldolase (1:3,500; Gentech), GFP (2:3,500; Roche), and histone H3 (1:10,000; Abcam) were incubated overnight at 4°C in blocking buffer. After three washes with 0.1% PBS-Tween 20 (Sigma), appropriate secondary antibodies IRDye 680RD anti-rabbit (1:10,000) and IRDye 800CW anti-mouse (1:10,000) IgG were added to the blot for 1 h at room temperature in blocking buffer with final concentrations of 0.1% Tween 20 (Sigma) and 0.05% sodium dodecyl sulfate (1st Base). The membrane was washed three more times with 0.1% PBS-Tween 20 (Sigma) and once with PBS. Membranes were scanned with a LI-COR Odyssey CLx imager.

### Immunofluorescence assay and microscopy.

Imaging of fixed PfYTH.2-sandwich parasites was performed as follows. Parasites were smeared onto glass slides, air-dried, and subsequently fixed for 5 min with 4% paraformaldehyde. The slides were blocked with 1% bovine serum albumin (BSA) and permeabilized with 0.1% Tween 20 in 1× PBS at room temperature for 1 h. Slides were then incubated for 1 h at room temperature with mouse anti-GFP antibody (Roche, 1:200). The slides were washed for 15 min with 1× PBST after primary antibody treatment followed by a 1-h incubation at room temperature with secondary antibody (1:10,000, anti-mouse IgG Alexa Fluor-488) and Hoechst (2 μg/ml). Slides were washed with 1× PBST at room temperature. The coverslips were mounted using Fluoromount-G (SouthernBiotech).

The PfYTH.2 fusion protein and the mislocalizer in the PfYTH.2-sandwich^ML^ cell line were visualized on live parasites. Briefly, parasites were incubated with 4′,6-diamidino-2-phenylindole (DAPI) for 10 min, washed once with DPBS, mounted on microscope slides, and imaged on a Delta Vision Elite microscope (GE Healthcare). Image overlays were generated using Fiji (39).

### PfYTH.2 co-immunoprecipitation and protein mass spectrometry.

P. falciparum PfYTH.2-sandwich (*n* = 3 replicates) and wild-type P. falciparum 3D7 (*n* = 2 replicates as negative control) were cultured in standard growth medium, sorbitol synchronized, and treated equally for the whole experiment. After 36 h, the parasites were harvested after Percoll (Sigma P4937) enrichment, washed twice with RPMI medium, and cross-linked with 0.5 mM dithiobis(succinimidyl propionate) (DSP) (Thermo Fisher 22585) in PBS for 60 min at 37°C (40). The reaction was quenched with PBS containing 25 mM Tris-HCl. The cell pellets were lysed with RIPA buffer (10 mM Tris HCl [pH 7.5], 150 mM NaCl, 0.1% SDS, and 1% Triton X-100) containing protease and phosphatase inhibitor cocktail (Thermo Fisher 78440). The lysates were cleared by centrifugation at 16,000 × *g* for 10 min. Supernatants were incubated with anti-GFP magnetic beads (Chromotek gtd-10) overnight at 4°C.

The beads were washed five times with 1 ml RIPA buffer following five washes with 1 ml PBS and one wash with 1 ml 100 mM ammonium bicarbonate (Sigma 09830). The beads were reduced with 10 mM dithiothreitol (Sigma D9779), alkylated with 55 mM iodoacetamide (Sigma I1149), and subjected to on-bead digestion using 1 μg of trypsin (Thermo Fisher 90059). The resulting peptides were desalted using C_18_ cartridges (Thermo Fisher 89852), and protein mass spectrometry was performed as described above. Candidate PfYTH.2-interacting proteins were filtered by excluding those that were also found in the control samples (e.g., resulting from background binding to antibody-bead complexes), and candidates had to be present with ≥2 unique peptides in at least 2 of 3 replicates (Table S2 and Fig. S2D).

### Crosslinking PfYTH.2-RNA co-immunoprecipitation followed by RT-qPCR.

PfYTH.2-sandwich^ML^ parasites were synchronized to a 6-h window using three consecutive rounds of sorbitol treatments. For the RIP experiment, two biological replicates were collected for the knock-sideways (i.e., plus rapamycin [+RAP]) and (i.e., no rapamycin [−RAP]) control treatment. At 18 h postinfection, rapamycin at a final concentration of 250 nM was added to the knock-sideways group. Three hours later, parasites of the knock-sideways and control treatment were collected by centrifugation (2,200 rpm for 2 min), lysed in 0.075% saponin in DPBS (Thermo Fisher 14190-144), and pelleted by centrifugation at 3,220 × *g* for 5 min. The pellet was resuspended in 10 ml DPBS, and methanol-free formaldehyde (Thermo Fisher 28908) was added to a final concentration of 1%. The cells were incubated for 10 min at 25°C with gentle agitation before cross-linking was quenched by addition of glycine (final concentration 125 mM) and incubation with gentle agitation for another 5 min at 25°C. Cells were collected by centrifugation (3,220 × *g* for 5 min at 4°C), washed twice with ice-cold PBS, and snap-frozen.

For the entirety of the PfYTH.2-RNA co-immunoprecipitation, +RAP and −RAP samples were treated identically. For each sample, 25 μl Dynabeads protein G magnetic beads (Thermo Fisher 10004D) were washed twice with 1 ml RIPA buffer (50 mM Tris-HCl [pH 7.4], 100 mM NaCl, 1% IGEPAL CA-630, 0.1% SDS, and 0.5% sodium deoxycholate) and then resuspended in 1 ml RIPA buffer. One microliter anti-GFP antibody (Abcam ab290) was added to the beads and rotated for 2 h at 4°C to allow binding of the antibody to the beads. In the meantime, cell pellets (∼4 × 10^8^) were resuspended in 500 μl RIPA buffer, supplemented with cOmplete Mini EDTA-free proteinase inhibitor (Sigma 4693159001) and 200 U SUPERase·In RNase inhibitor (Thermo Fisher AM2694). The cells were lysed with 200 strokes in a prechilled 2 ml Dounce homogenizer and then sonicated for 5 cycles with settings of 30-s on and 30-s off in a BioRuptor Pico sonicator. The cell lysate was cleared of debris by centrifugation (16,000 × *g* for 10 min at 4°C). Following incubation, the antibody (Ab)-bead mixture was washed twice with 1 ml RIPA buffer and then added to the cell lysate and incubated with rotation overnight at 4°C. The beads were collected on a magnet, and the supernatant was discarded. The beads were stringently washed by resuspension and magnet separation twice with RIPA buffer, once with low-salt wash buffer (20 mM Tris-HCl [pH 8], 150 mM NaCl, 2 mM EDTA [pH 8], 1% Triton X-100, and 0.1% SDS), once with high-salt wash buffer (20 mM Tris-HCl [pH 8], 500 mM NaCl, 2 mM EDTA [pH 8], 1% Triton X-100, and 0.1% SDS), and once with LiCl wash buffer (10 mM Tris-HCl [pH 8], 250 mM LiCl, 1 mM EDTA [pH 8], 0.5% IGEPAL CA-630, and 0.5% sodium deoxycholate). The protein-RNA complexes were eluted from the beads by incubation with 205 μl elution buffer containing 100 pg of a spike-in transcript for 30 min at 70°C. The beads were collected on a magnet, and 200 μl of the eluate was mixed with 600 μl of TRIzol LS reagent (Thermo Fisher 10296028). RNA was extracted according to the manufacturer’s instructions and precipitated by ethanol precipitation. cDNA was generated using the Superscipt VILO cDNA synthesis kit (Thermo Fisher 11754050) according to the manufacturer’s protocol. We selected target transcripts that were previously identified to have at least one m^6^A peak at the trophozoite stage (12). Target transcripts were amplified in technical triplicates using Power Sybr green PCR master mix (Thermo Fisher 4367659) and the primers listed in Table S4 on a Bio-Rad CFX qPCR machine. A no-reverse transcription (RT) control (substitution of RT during cDNA synthesis with H_2_O) and a no-template control (no cDNA added during qPCR amplification) were included in all experiments. Threshold cycle (*C_T_*) values were normalized to the spike-in transcript (i.e., Δ*C_T_*) in each sample, and relative enrichment for each transcript (i.e., ΔΔ*C_T_*) was calculated by subtraction of the −RAP Δ*C_T_* from the +RAP Δ*C_T_* in the corresponding control and treatment samples.

### PfYTH.2 gradient purification.

PfYTH.2-sandwich parasites were synchronized to a 6-h window by three consecutive sorbitol treatments. At 18 hpi, parasites were collected by centrifugation (2,200 rpm for 2 min) and lysed in 0.075% saponin in DPBS (Thermo Fisher 14190-144). The parasite cell pellet was washed twice in cytoplasmic lysis buffer (CLB; 10 mM NaCl, 5 mM MgCl_2_, 20 mM Tris HCl [pH 8], and 1% Triton X-100) and snap-frozen at −80°C.

The pellet from ∼10^9^ parasite cells was resuspended in 500 μl CLB supplemented with 200 U SUPERase·In RNase inhibitor (Thermo Fisher AM2694), 100 μg cycloheximide (Sigma C4859-1ML), and cOmplete Mini EDTA-free proteinase inhibitor (Sigma 4693159001). The cells were lysed in a 2-ml prechilled Dounce homogenizer, and nuclei and cell debris were pelleted by centrifugation (16,100 × *g* for 10 min at 4°C). The protein concentration of the supernatant was measured using the Pierce *A*_660_ reagent (Thermo Fisher 22660) on a NanoDrop (Thermo Fisher). The protein lysates were layered on top of a linear sucrose gradient (10% to 50%) prepared in ultracentrifuge tubes (Beckman Coulter 344059) and fractionated by centrifugation (3 h at 39,000 rpm at 4°C) in a Beckmann coulter ultracentrifuge using a SW-41 swinging bucket rotor. The gradient was fractionated into 10 different fractions on a fraction collector, continuously measuring the 260-nm absorbance, and stored at −80°C.

Proteins were precipitated by adding 10% (vol/vol) trichloroacetic acid to the sucrose fractions and incubating on ice for 4 h. Proteins were pelleted by centrifugation (18,000 × *g* for 10 min at 4°C) and washed thrice with ice-cold acetone. The pellet was air dried and resuspended in 10 μl 4× NuPage LDS sample buffer (Thermo Fisher NP0007). For Western Blot analysis, the samples were diluted to a 2× NuPage sample buffer concentration using 10 μl RIPA buffer (50 mM Tris-HCl [pH 7.4], 100 mM NaCl, 1% IGEPAL CA-630, 0.1% SDS, and 0.5% sodium deoxycholate). Proteins were denatured for 10 min at 70°C, run on a NuPage 4% to 12% bis-Tris gel using morpholinepropanesulfonic acid (MOPS) running buffer at 150 V and transferred to a PVDF membrane using a Trans-Blot Turbo transfer pack (Bio-Rad 1704157) with the Trans-Blot Turbo transfer system (Bio-Rad). The membrane was blocked for 1 h in 1% milk in 0.1% Tween 20 in PBS (PBST). GFP-tagged PfYTH.2 was detected using rabbit anti-GFP (ChromoTek [PAPG1]; 1:1,000 in 1% milk-PBST) primary antibody, followed by donkey anti-rabbit (GE number NA934-1ML) secondary antibodies conjugated to HRP (1:5,000 in 1% milk-PBST). The HRP signal was developed using the SuperSignal West Pico chemiluminescent substrate (Thermo Fisher 34580) and imaged with a ChemiDoc XRS+ (Bio-Rad).

### Ribosome profiling.

PfYTH.2-sandwich^ML^ parasites were synchronized to a 6-h window by three consecutive sorbitol treatments as described above. At 18 hpi, rapamycin was added at a final concentration of 250 nM to the treatment (i.e., knock sideways) group. Three hours later, parasites were collected by centrifugation (2,200 rpm for 2 min) and lysed in 0.075% saponin in DPBS (Thermo Fisher 14190-144). For the ribosome profiling samples, the parasite cell pellet was washed twice in cytoplasmic lysis buffer (CLB; 10 mM NaCl, 5 mM MgCl_2_, 20 mM Tris HCl [pH 8], and 1% Triton X-100) and snap-frozen at −80°C. For the ribosome profiling control (no rapamycin) and treatment (with rapamycin) groups, two replicates with ∼10^9^ parasites were collected. In parallel, 5 × 10^7^ parasite cells from the same replicate samples were resuspended in 700 μl QIAzol following saponin lysis and stored at −80°C for mRNA sequencing.

Cell lysis was performed as for the PfYTH.2 gradient purification described above but with an additional partial RNA treatment to digest polysomes into individual monosomes. Briefly, the cell pellets were resuspended in 500 μl CLB supplemented with 200 U SUPERase·In RNase inhibitor (Thermo Fisher AM2694), 100 μg cycloheximide (Sigma C4859-1ML), and cOmplete Mini EDTA-free proteinase inhibitor (Sigma 4693159001). Cells were lysed in a 2-ml prechilled Dounce homogenizer and cleared from nuclei and cell debris by centrifugation (16,100 × *g* for 10 min at 4°C). The protein concentration of the supernatant was measured using the Pierce *A*_660_ reagent (Thermo Fisher 22660) on a NanoDrop (Thermo Fisher), and ∼600 ng of protein lysate was subjected to partial RNase digestion using 100 U RNase I (Thermo Fisher AM2294) in a thermomixer (5 min at 37°C and 300 rpm). The RNase-treated protein lysates were layered on a linear sucrose gradient (10% to 50%) and fractionated by ultracentrifugation (3 h at 39,000 rpm at 4°C) as described above.

The sucrose gradient was fractionated, and the 80S monosome fraction was detected by the 260-nm absorbance, collected in a microcentrifuge tube, and immediately mixed with TRIzol LS reagent (Thermo Fisher 10296010). The aqueous RNA phase was separated according to the manufacturer’s instructions, and RNA was precipitated by isopropanol precipitation and resuspended in 12 μl RNase-free water. To select the ribosome protected fragments (RPF), 10 μl of the RNA sample was denatured using 10 μl 2× Novex Tris-borate-EDTA (TBE)-urea sample buffer (Thermo Fisher LC6876) and run on a 15% Novex TBE-urea gel (Thermo Fisher EC6885BOX). As size marker, 25-nt and 32-nt RNA oligonucleotides were run on the same gel. The gel was stained for 30 min in 50 ml RNase free 1× TBE buffer supplemented with 5 μl Sybr gold (Thermo Fisher S11494), and the RPF fraction (25 nt to 32 nt) for each sample was cut from the gel using razor blades. Gel slices were crushed in 1.5-ml tubes and resuspended in 300 μl gel extraction buffer (0.3 M sodium acetate [NaOAc], 1 mM EDTA, and 0.25% [vol/vol] SDS). The solution was frozen for 30 min on dry ice and then left at 25°C overnight in a thermomixer at 500 rpm. RPFs were extracted from the solution by ethanol precipitation. To allow for adaptor ligation, RPF 3′ ends were dephosphorylated using T4 PNK (New England BioLabs M0201S) for 30 min at 37°C, followed by the addition of 10 mM ATP and another 30 min incubation at 37°C for RPF 5′ phosphorylation. RPF fragments were purified using ethanol precipitation, and small RNA libraries were prepared using the NEBNext Multiplex small RNA library prep kit for Illumina (New England BioLabs E7300) according to the manufacturer’s instructions.

### RNA-sequencing.

Total RNA from the two replicates of treatment and control PfYTH.2-sandwich^ML^ collected in parallel with the ribosome profiling samples were extracted as described above. Poly(A) RNA was enriched using the Dynabeads mRNA purification kit (Thermo Fisher 61006). RNA sequencing libraries were prepared using the Illumina TruSeq stranded RNA library prep kit (Illumina RS-122-2101) according to the manufacturer’s instructions with slight modifications. To account for the AT richness of cDNA fragments, we used the KAPA Hifi polymerase (Roche 07958846001) at the library amplification step. The libraries were sequenced on an Illumina NextSeq 500 platform with a 2 by 150-bp paired-end read layout.

### Analysis of translational efficiencies.

Raw image files were converted to fastq sequence files using Illumina’s bcl2fastq (v2.19), and adaptors and low-quality read ends were trimmed using Trimmomatic (41). The mRNA and RPF sequencing reads for the two replicates of ±RAP PfYTH.2-sandwich^ML^ samples were mapped to the P. falciparum genome, and PCR duplicates were removed using SAMtools (42). For the mRNA samples, only read alignments with a MAPQ quality score of ≥20 were retained. Raw gene count values of the mRNA (strand specific) and ribosome footprints (non-strand specific) were calculated in R using the package htseq-count (43). Gene count tables were merged and the fragments per kilobase of exon per one million mapped reads (FPKM) were calculated in R using the fpkm() function within the DESeq2 package (44). FPKM-derived translational efficiencies were calculated as FPKM(RPF)/FPKM(mRNA). ΔTE values were calculated using DESeq2 integrated in the deltaTE workflow (45) from raw mRNA and RPF values.

### Phylogenetic analysis of YTH proteins.

Full-length protein sequences of PfYTH.1, PfYTH.2, and representative proteins from model eukaryotes were downloaded from UniProt, and multiple-sequence alignments were performed using mafft (46) with default settings. Gaps were removed with trimAl (47), and the best phylogenetic model (i.e., WAG) was calculated using ProtTest3 (48). A maximum likelihood phylogenetic tree was constructed using MEGA (v7) (49) with the WAG model and 1,000 bootstrap replicates. The bootstrap consensus trees were visualized in FigTree (v1.4.3, http://tree.bio.ed.ac.uk/software/Figtree/).

### Statistical analysis.

All statistical analyses were performed in R (50). To test for a normal distribution of the data, the Shapiro-Wilk normality test was used. To test for significance between two groups, a two-sided independent-samples *t* test or two-sided Mann-Whitney *U* test was performed where indicated. Gene ontology enrichments were calculated using the build-in tool at https://plasmoDB.org. Correlations were calculated in R using function cor() with default settings (i.e., calculation of Pearson correlation coefficient *r*).

### Data availability.

Ribosome profiling and mRNA sequencing data were deposited under NCBI BioProject PRJNA659894. RNA modification mass spectrometry, proteomics data of the RNA oligonucleotide pulldown, and the PfYTH.2 co-immunoprecipitation data are available at https://chorusproject.org/ with accession number 1694.
