# Supplementary figures and images for: Functional Characterization of the m6A-Dependent Translational Modulator PfYTH.2 in the Human Malaria Parasite
Source: mBio. 2021 Apr 27;12(2):e00661-21. doi: 10.1128/mBio.00661-21 (PMC8092261; doi:10.1128/mBio.00661-21)

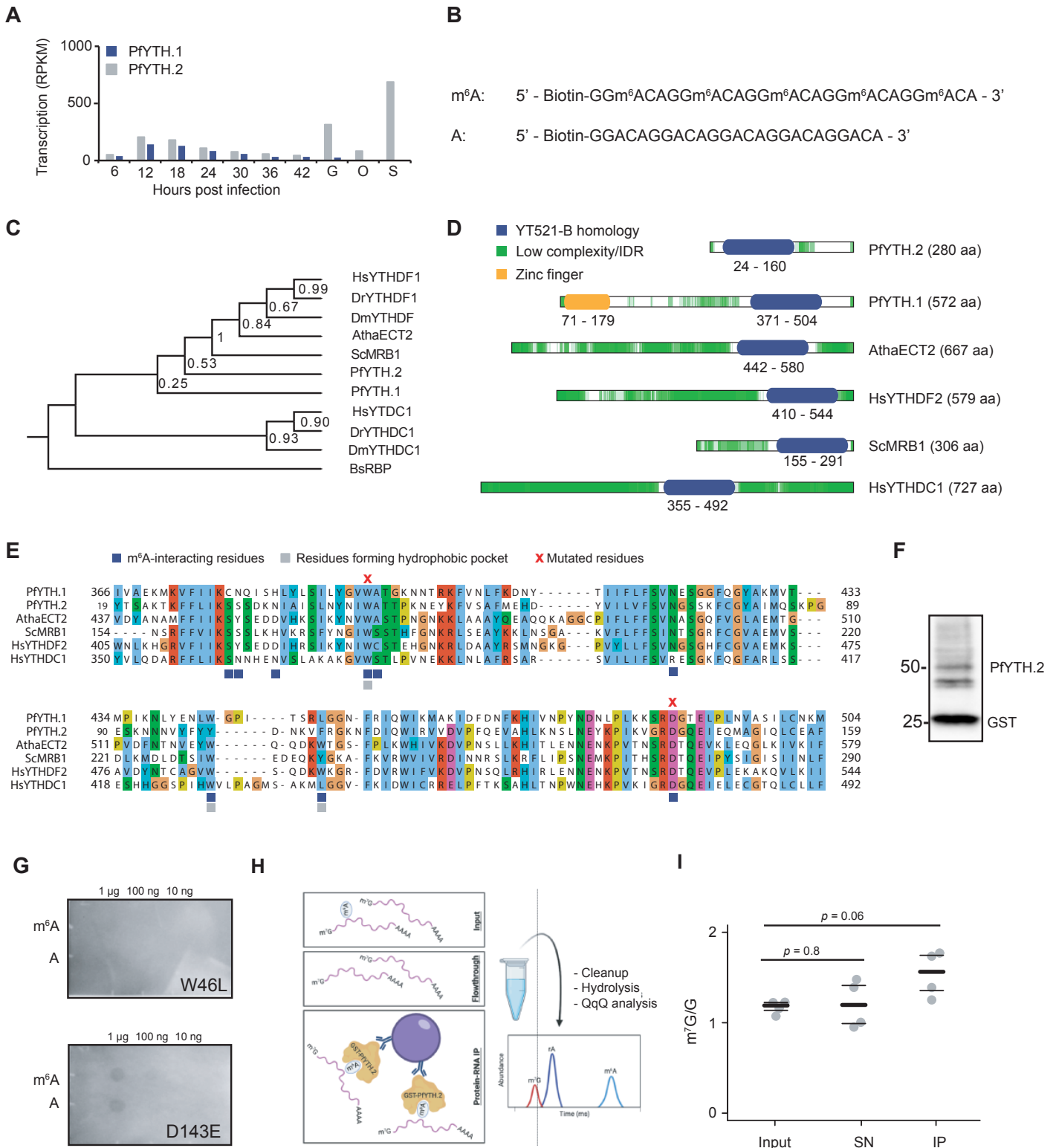

Supplement: FIG S1 [file mBio.00661-21-sf001.pdf]

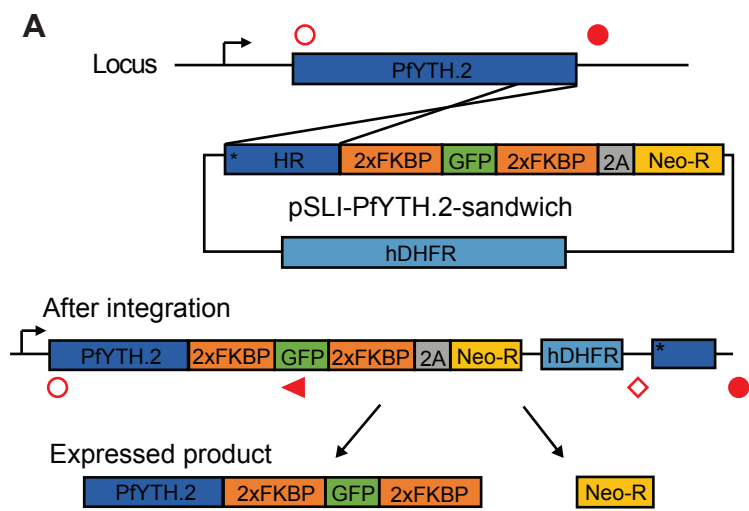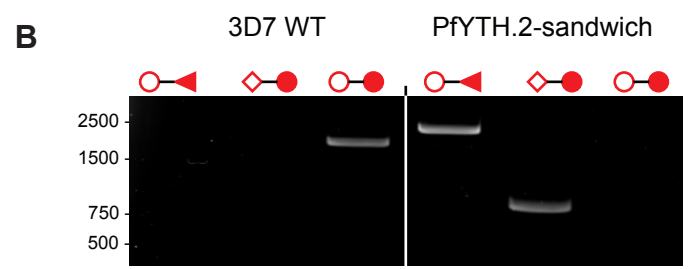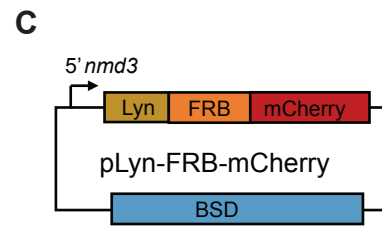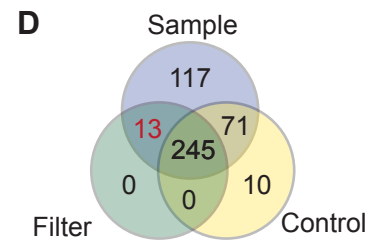

Supplement: FIG S2 [file mBio.00661-21-sf002.pdf]

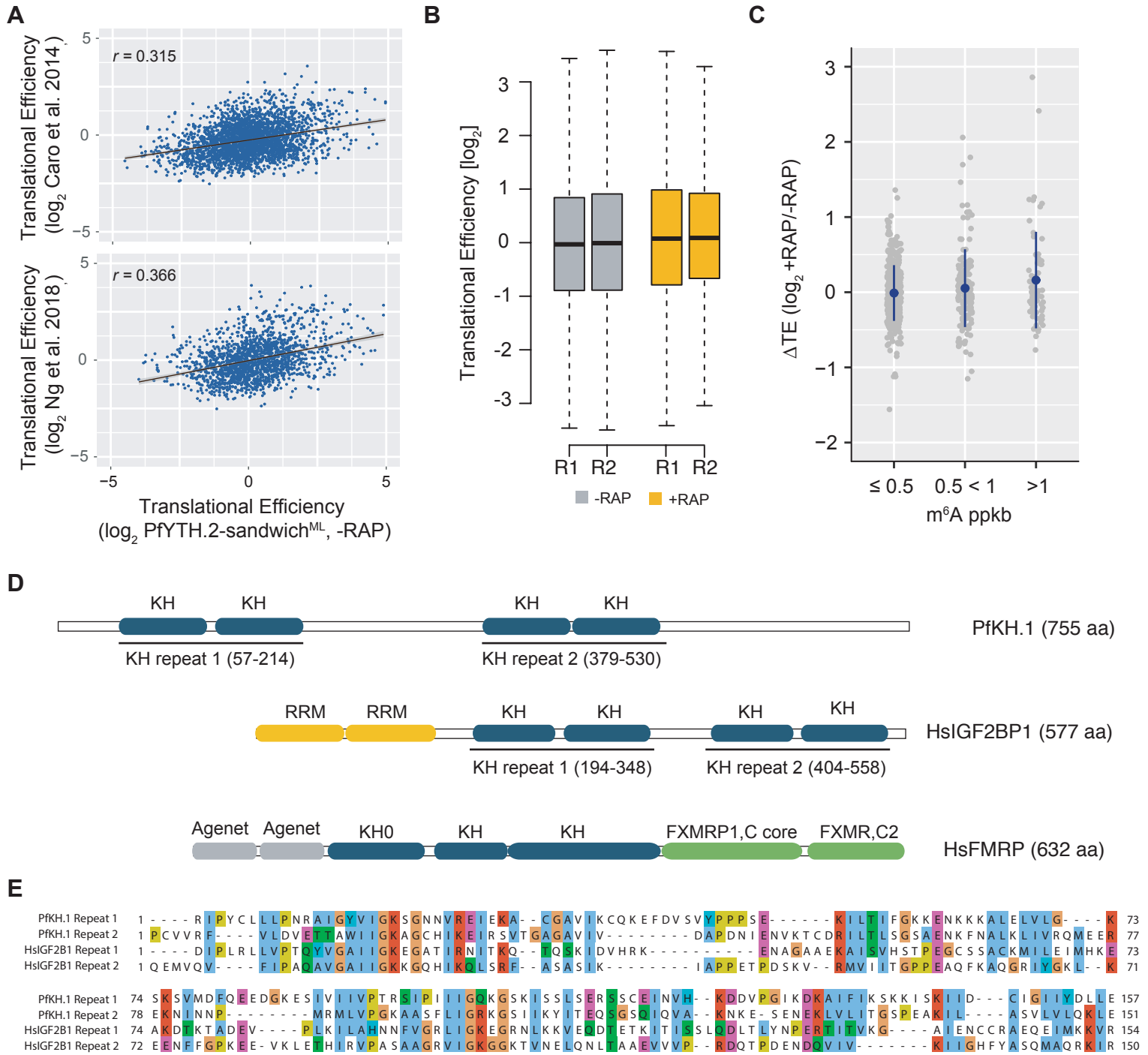

Supplement: FIG S3 [file mBio.00661-21-sf003.pdf]
